# Prototype of an App Designed to Support Self-Management for Health Behaviors and Weight in Women Living With Breast Cancer: Qualitative User Experience Study

**DOI:** 10.2196/48170

**Published:** 2024-12-20

**Authors:** Phillippa Lally, Christine N May, E Siobhan Mitchell, Meaghan McCallum, Andreas Michaelides, Abigail Fisher

**Affiliations:** 1 School of Psychology University of Surrey Surrey United Kingdom; 2 Academic Research Noom Inc New York, NY United States; 3 Department of Behavioural Science and Health University College London London United Kingdom

**Keywords:** breast cancer, self-management, app, health behaviors, weight, prototype, user experience, development, application, coaching, peer support, oncology

## Abstract

**Background:**

Accessible self-management interventions are required to support people living with breast cancer.

**Objective:**

This was an industry-academic partnership study that aimed to collect qualitative user experience data of a prototype app with built-in peer and coach support designed to support the management of health behaviors and weight in women living with breast cancer.

**Methods:**

Participants were aged ≥18 years, were diagnosed with breast cancer of any stage within the last 5 years, had completed active treatment, and were prescribed oral hormone therapy. Participants completed demographic surveys and were asked to use the app for 4 weeks. Following this, they took part in in-depth qualitative interviews about their experiences. These were analyzed using thematic analysis.

**Results:**

Eight participants (mean age, 45 years; mean time since diagnosis, 32 months) were included. Of the 8 participants, 7 (88%) were white, 6 (75%) had a graduate degree or above, and 6 (75%) had stage I-III breast cancer. Four overarching themes were identified: (1) Support for providing an app earlier in the care pathway; (2) Desire for more weight-focused content tailored to the breast cancer experience; (3) Tracking of health behaviors that are generally popular; and (4) High value of in-app social support.

**Conclusions:**

This early user experience work showed that women with breast cancer found an app with integrated social and psychological support appealing to receive support for behavior change and weight management or self-management. However, many features were recommended for further development. This work is the first step in an academic-industry collaboration that would ultimately aim to develop and empirically test a supportive app that could be integrated into the cancer care pathway.

## Introduction

An estimated 55,200 people in the United Kingdom and 281,550 in the United States are diagnosed with breast cancer each year, and in 2021, breast cancer became the most common cancer globally [1-3]. The overall 5- and 10-year survival rates are estimated at 85% and 75%, respectively [3], indicating that very large numbers of people are living with and beyond their diagnosis. However, breast cancer and its associated treatments can lead to multiple immediate and long-term side effects [4,5]. Physical activity, diet, and maintenance of healthy weight play important roles in the management of breast cancer [5], and many women desire to receive support for these as part of their cancer care [6,7]. However, given the pressure on health care systems and the other barriers that health care professionals face in delivering behavioral interventions in practice [8,9], accessible self-management interventions are required.

Smartphones have the potential for the delivery of scalable supportive interventions, and ownership is continually increasing. For example, between 2011 and 2021, the proportion of US adults who own smartphones increased from 35% to 85% [10]. There are also examples of how, following substantial user development and empirical testing, a smartphone-delivered intervention can become part of the recommended clinical referral pathway (eg “Sleepio” for insomnia in the United Kingdom in 2022 [11]). Many women with breast cancer want to play an active role in the management of their health, and when surveyed, 68% were interested in information delivered via an app or website [12]. However, alongside this is the desire for peer support from others who have experienced cancer [13], with evidence that this can facilitate positive behavioral change [14]. Systematic reviews have highlighted a number of existing apps designed for cancer self-management [15-18]. A meta-analysis of 30 randomized controlled trials of apps found that they improved quality of life and psychological symptoms among people living with cancer [19]. However, few apps incorporate combined support for physical activity, diet, and weight management or integrated social support [15-18]. A review of app-based interventions for supporting lifestyle or healthy behavioral change in people living with cancer, which was published in 2021, included studies of 17 apps designed to promote diet, physical activity, or mental health in people living with cancer. The majority of these apps were explored in pilot studies, qualitative interviews (one from our group described below), or descriptive studies [20], highlighting that there is insufficient evidence to recommend any particular app. In addition, few apps for breast cancer management have been developed or user-tested by collaborative academic and industry partners, a highlighted limitation of the current field [16,18]. An app-store scoping review found 151 apps aimed at people living with cancer (broadly covering the areas of information provision, organizing cancer care, interacting with others, and cancer management). Most were developed commercially, and only 13 of these apps were developed in partnership with organizations [21]. Such a collaboration is important for quality, optimization, empirical testing, and sustainability.

A key early step in the research and development of a supportive care app is appropriate testing with target users. In a focus group study gathering the views of 35 people living with cancer on app features (using screenshots for discussion), participants highlighted a preference for a positive casual tone, tools to support goal attainment, a “prescription” for physical activity, and individual tailoring [22]. A study of 11 people living with cancer, which was designed to explore their experiences of using a physical activity app for 6 weeks, identified a number of valued app features, including tailoring and social support [23]. We also asked people with breast (n=8), prostate (n=16), or colorectal cancer (n=8) to download and use physical activity apps over a 2- to 3-week period and then conducted in-depth qualitative interviews on their experiences [13]. This provided not only invaluable details of how users felt about the apps they tested but also a far richer understanding of the factors that are important to people living with and beyond cancer than would have been gained from interviews on hypothetical app use. This work subsequently informed the design and funding of a pilot randomized controlled trial of a physical activity app for people with breast, prostate, or colorectal cancer [24].

The aim of this study was to use a similar methodology to gather user experience data of a prototype app with built-in peer and coach support designed to support the management of health behaviors and weight in women living with breast cancer. The information provided in this study will highlight which app components are most valued by potential users, feed into further development of the app, and provide valuable information on what women with breast cancer want from digital support in general.

In this manuscript, we will describe the study design, including the app content, and will then present details of the participant demographics, their app usage during the study, and the themes derived from a qualitative analysis based on their interviews. We will then discuss these findings and what they add to the literature.

## Methods

### Design

This was a qualitative user experience study where participants were asked to complete a brief online survey, use the app (Healthy Habits after Cancer: HHC) for 4 weeks, and then participate in qualitative interviews. The study was conducted between June and August 2021. Our previous work suggested that 8-10 participants with 1 cancer type trying a single app would provide meaningful qualitative user experience data [13].

### Participants

Recruitment was conducted in the United States through the Living Beyond Breast Cancer nonprofit organization via email and social media advertising. Members of the organization who were interested were invited to complete an online screening questionnaire to assess eligibility. Participants were eligible if they were female, were aged ≥18 years, were diagnosed with breast cancer of any stage within the last 5 years, had completed active cancer treatment (eg, chemotherapy, surgery, or radiotherapy) at least 1 month previously, were currently prescribed oral hormone therapy (eg, tamoxifen; medication tracking was a feature of the app we were looking for feedback on), were not pregnant or planning to become pregnant in the next 5 months, were not diagnosed with an eating disorder, had a BMI of >18.5, and owned a smartphone with the iOS operating system as the prototype was built for iOS only. The exclusion criteria were any surgery planned within 6 weeks of study enrolment, current treatment for a second primary cancer (ie, cancer of another organ, not due to breast cancer metastasis, with the exception of nonmelanoma skin cancers), inability to understand spoken or written English, and presence of a diagnosed physical or mental health condition that would impact the ability to participate. If there was any doubt over whether individuals met these criteria, it was recommended that they discuss participation with their clinical care team before enrolling; however, this was not required for any of the participants. Those deemed eligible were invited to complete the online informed consent form and baseline survey before they were given access to the HHC app.

Participants received free access to the HHC app for the duration of the study and were provided with a US $20 gift card at the end of the study.

### Ethical Considerations

Ethical approval was provided by the Advarra Institutional Review Board (approval number: Pro00055029), and all participants provided informed consent for the study. The Advarra Institutional Review Board is a centralized ethics review board that operates in compliance with US federal regulations and the ethical principles underlying the involvement of human subjects in research [25].

### HHC App

**Figure 1:**
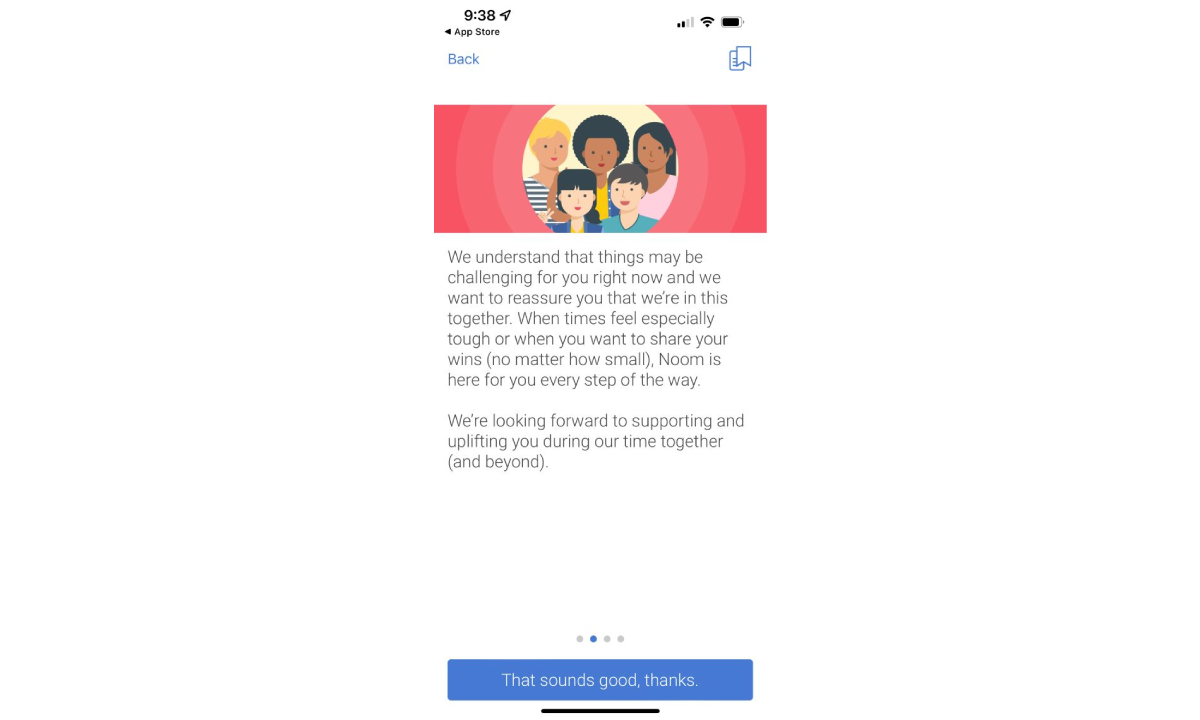
Screenshot of the Noom Health Habits after Cancer app: introductory page.

**Figure 2:**
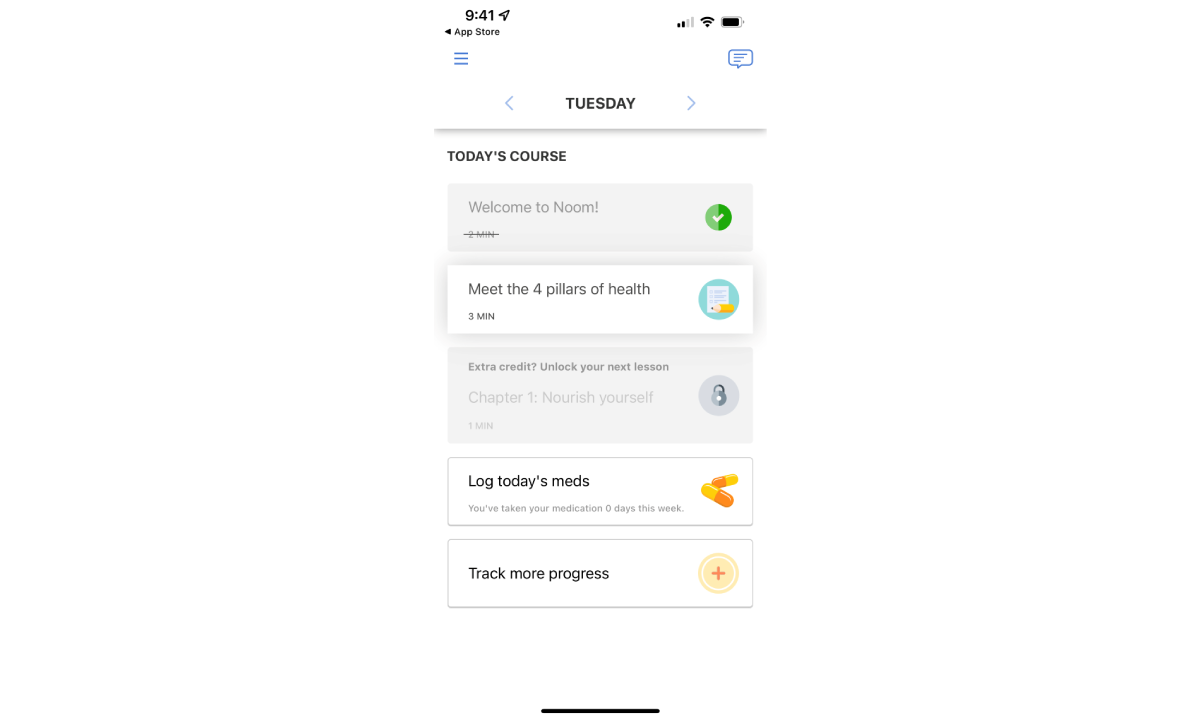
Screenshot of the Noom Healthy Habits after Cancer app.

**Figure 3:**
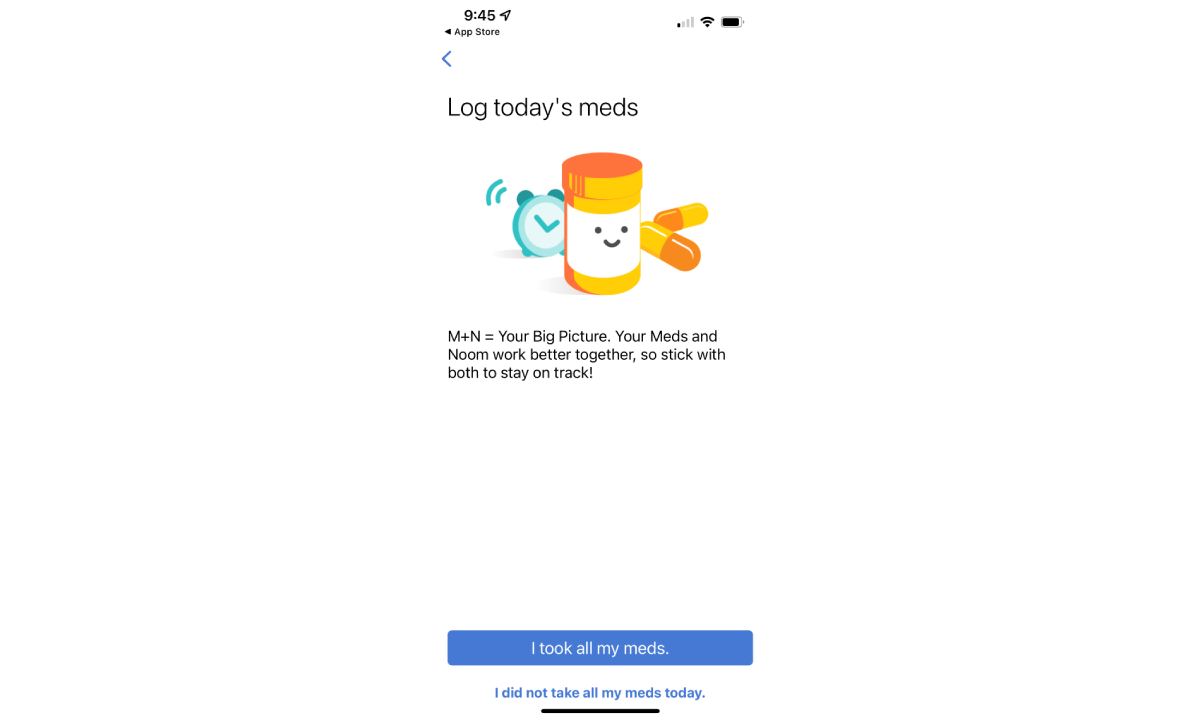
Screenshot of the Noom Health Habits after Cancer app: medication tracker.

**Figure 4:**
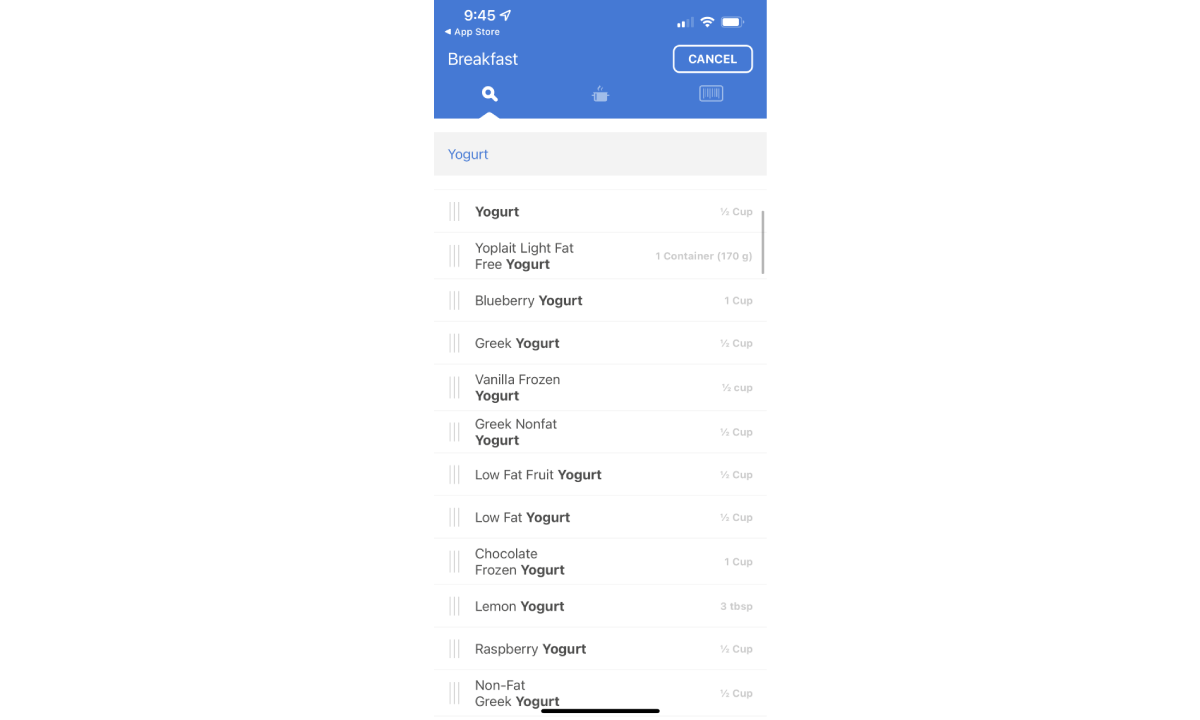
Screenshot of Noom Health Habits after Cancer app: diet tracker.

Following preliminary discussions about the content between researchers at Noom and University College London, the HHC prototype was created by Noom, who developed a popular weight loss program for the general population and planned to explore the development of a supportive app for women living with breast cancer. The HHC app was based on psychological behavior change principles from cognitive behavioral therapy (CBT) [26,27], acceptance and commitment therapy (ACT) [28], and dialectical behavior therapy (DBT) [29], as well as the guidelines of the American Cancer Society and World Cancer Research Fund. The key components of the app are summarized in Table 1. Screenshots of example app pages are shown in Figures 1-4.

**Table 1:** Key content of the Healthy Habits after Cancer app.

Intervention component	Details
Curriculum (articles)	Participants are encouraged to read short daily articles in the app. These address topics, including the 4 pillars of health: nourishment (focusing on mental and behavioral habits), self-care (prioritizing your own well-being), positive experiences (eg, focusing on enjoyment and gratitude), and support (eg, reframing negative thoughts).
Calorie targets	Calorie targets are set based on basal metabolic rate and logged exercise.
Meal logging	Participants are asked to log all the food they eat via the app, which calculates their calorie intake and displays this against their target.
Weight logging	Participants set their own weight target and are asked to log their weight weekly in the app.
Physical activity	Participants can set their own target or choose automatic adjustable step targets (initially set at 2000 steps a day and increased or decreased over time based on tracked steps). Participants can link an activity tracker to the app or manually enter their steps.
Recording of water intake	Participants set their own water intake target and are asked to log their water intake in the app.
Medication logging and reminders	Participants are asked to log their medication use in the app daily and are sent medication reminders.^a^
Individual one-to-one coaching	A one-to-one coach communicates with participants via in-app messaging. Participants have access to their coach chat at all times. The coach responds to participant messages within 24 hours or less.
Group support	An in-app closed group of other women with breast cancer and a group coach are available for discussions via an in-app chat area.

^a^In the prototype, reminders are sent via text messages outside the app rather than as pop-ups within the app.

During the first week of the study, participants were introduced to the HCC app, their one-to-one coach, and their support group via the app and were given unlimited free access to the program. They were not instructed to use the app a certain amount but were told that there were daily tasks and articles provided. The coaches had been through Noom’s intensive training program. The training is approximately 2 weeks in length, and the philosophy of the coach training is centered on motivational interviewing and active listening. Noom health coaches come from a variety of backgrounds (ie, nutrition, exercise physiology, and mental health professionals). The Noom health coach who worked on this study specifically was a licensed marriage and family counselor who worked within the scope of the Noom health coach role, and had experience working with cancer patients. Coaches used a secure dashboard to monitor participant app use, physical activity, meal logging patterns, dietary intake, and weight, which is used to increase engagement and motivation, and communicated with participants via in-app asynchronous messaging.

A group coach moderated the in-app social support groups by posting content to promote discussion and responding to user posts or comments. During this study, given the small sample size, the group involving this sample was merged with another breast cancer survivor group mid-study to encourage more group engagement and keep conversations more continuous. The other group was from a study Noom was running concurrently with the Dempsey Cancer Center, Maine, which was looking at a group of women diagnosed with breast cancer (stage I, II, or III) within the last 5 years and who had completed active treatment (eg, surgery, chemotherapy, or radiation) at least 6 months ago. They had access to the app for 6 months.

### Sociodemographic Variables

At baseline, participants completed an online survey providing date of birth (to calculate age in years at the date of survey completion), race, marital status, employment, education level, date of diagnosis (used to calculate months since diagnosis), cancer stage at diagnosis, height and weight (used to calculate BMI), and details of cancer treatments.

### Qualitative Interviews

Interviews were guided by a semistructured schedule designed to explore general experiences of cancer and weight management as well as to probe about specific elements of the HHC app. The interview schedule is provided in Multimedia Appendix 1. One-to-one telephone or video-call interviews were conducted by a member of the Noom research team with training in qualitative interviewing (author MM). MM had no prior relationship with the participants interviewed. Interviews were audio recorded and transcribed verbatim, and the information was deidentified (removing any reference to names or locations).

### App Usage

App usage was recorded as the total number of in-app engagements each week. An in-app engagement is defined as reading an article; messaging a coach; interacting with the support group; or logging a meal, water intake, weight, medication, or exercise.

### Analyses

Survey analyses were conducted using descriptive statistics. App engagement was assessed through the number of participants using the app at all each week, as well as the average number of engagements each week across those participants who engaged at all.

Qualitative analyses were conducted in NVivo software (QSR International) using reflexive thematic analysis following the 6 phases outlined by Braun and Clarke [30,31]. First, 2 researchers (AF and PL) familiarized themselves with the data by reading and rereading all transcripts and taking notes, and then independently coded the same randomly selected transcript, met to discuss similarities and differences, and generated an initial set of codes that was used to code the rest of the transcripts, with scope to add new codes as required and conduct regular meetings to discuss ongoing findings. Potential themes and subthemes were generated by reviewing all codes. These were then presented back to the wider research team, reviewed, named, and finalized for presentation in this paper (along with illustrative quotes). A deductive and inductive approach to analysis was taken with the aims of understanding the experience of using the HHC content and gathering data on the wider experience of breast cancer and the desired content from a digital intervention.

## Results

### Participants

It was not possible to know how many potential participants viewed the recruitment advertisements, but 42 expressed interest, 15 signed the consent form, 13 enrolled in the HHC program, and 8 used the app for 4 weeks and took part in interviews. Participant characteristics are presented in Table 2. Of the 8 participants, 7 (88%) were white and 1 (22%) was mixed race. All were married, and the majority (6/8, 75%) had a graduate degree or above.

**Table 2:** Participant characteristics.

Characteristic		Value (N=8)
Age (years), mean (SD)		45 (8)
**Race, n (%)**		
	White	7 (88)
	Mixed race	1 (22)
**Education, n (%)**		
	High school diploma or GED^a^	2 (25)
	Graduate degree or above	6 (75)
**Marital status, n (%)**		
	Married	8 (100)
**Employment, n (%)**		
	Employed full time	5 (62.5)
	Employed part-time	3 (37.5)
**Cancer stage at interview, n (%)**		
	Localized	6 (75)
	Metastatic	2 (25)
Months since diagnosis, mean (SD)		32 (14)
**Chemotherapy, n (%)**		
	Yes	6 (75)
	No	2 (25)
**Radiotherapy, n (%)**		
	Yes	6 (75)
	No	2 (25)
BMI (kg/m^2^), mean (SD)^b^		29 (5)

^a^GED: general educational diploma.

^b^Two participants did not report BMI.

All 8 participants were engaged for the first 3 weeks, and 7 of the 8 participants were engaged for all 4 weeks. The number of engagements each week is shown in Table 3.

**Table 3:** App engagement over the 4-week study (N=8).

Variable	Week			
	1	2	3	4
				
Number of engaged participants	8	8	8	7
Average number of total weekly in-app engagements	77.0	54.6	64.9	44.1

Themes and subthemes are presented in Textbox 1. Four overarching themes were identified: (1) Support for providing an app earlier in the care pathway; (2) Desire for weight-focused content tailored to the breast cancer experience; (3) Tracking of health behaviors that are generally popular; and (4) High value of in-app social support.

**Themes and subthemes.**

**1. Support for providing an app earlier in the care pathway**

- More time during active treatment

- Thirst for knowledge about health in early stages

- Later in the pathway already found a community

- Recommendations from oncologists (most powerful)

**2. Desire for weight-focused content tailored to the breast cancer experience (“It wasn’t what I was expecting”)**

- Style of articles was enjoyed

- Too much focus on emotional content, not enough on weight (loss)

- Desire for increased tailoring to the breast cancer experience

**3. Tracking of health behaviors** **that are generally popular**

- Meal logging and color coding of diet were popular

- Water or fluid tracking was useful for the cancer community

- Physical activity tracking was very useful, but the step target was too low

- Increased prominence of physical activity preferred

- Medication adherence feature not used

**4. High value of in-app social support**

- Coaches provided accountability and increased engagement

- Groups were popular

- Mixed views on including early stage and metastatic disease together

### Support for Providing an App Earlier in the Care Pathway

Participants expressed a strong desire for support (especially around weight loss). They had completed active curative treatment when they participated in the study and strongly felt that this app would be more appropriate for those still on active treatments such as chemotherapy and radiotherapy.

I feel like it would have been really helpful for me during chemo and my active treatment.

age 40-49 years; localized disease

While some suggested they would like to have had access to the app at diagnosis, most discussed that they would still have been too shocked at this point to use it.

...right at diagnosis my brain was completely scrambled with the whirlwind of everything that was going on, I’m not sure at diagnosis I would have had the focus to give it any kind of attention.

age 30-39 years; localized disease

#### More Time During Active Treatment

A key reason women felt that early in the pathway would be a good time was the additional time they had while on active treatment due to, for example, stopping work and social activities. Although they often felt fatigued on treatment, they felt that they would have had the mental capacity to engage with many of the app components and to start to think about health and behaviors.

I may have been able to use it more, like, when I was more active in treatment because my life was a little bit less busy, if you will…And I think I might have engaged more in the 1:1, in the group setting, because I think that would have helped fill areas that, because I was not working quite as much -- you know...

age 40-49 years; localized disease

I mean radiation therapy is exhausting -- if you start on Monday, by Friday you can barely walk upstairs -- but you have enough bandwidth to start thinking about your health.

age 40-49 years; localized disease

...chemo definitely (be)cause you sit in a chair for hours with nothing to do, that would be a good like positivity booster and things like that while you’re sitting there.

age 40-49 years; localized disease

#### Thirst for Knowledge About Health in Early Stages

Some participants described a “sweet spot” once treatment had started where they were keen to gather as much information as possible and take ownership of their health.

I think earlier in my cancer journey, when I was so focused on losing weight, I probably would have perhaps asked my coach, like “hey, I'm looking for more nutrition content, or this, or that’ and just really explored what could have been available or what resources she could have provided for me, but I didn’t push on that because I am much further along in my journey, so I think for earlier-diagnosed people, you’re so thirsty for information.

age 50-59 years; metastatic disease

Others described a desire to learn more about interactions between health behaviors and active treatment side effects (like which dietary factors might alleviate or enhance diarrhea or nausea) as these were happening.

I want to know like the changes my body’s going through and how I can change certain aspect of my life whether it is my diet or exercise or anything to that effect that would improve my quality of life or at least be aware of potential side effects and things of that nature.

age 30-39 years; localized disease

#### Later in the Pathway Already Found a Community

Some participants discussed that by the time they had completed active treatment, they had found a community of other breast cancer patients and that if the app had come earlier in the pathway when they were still seeking peer support, they would have engaged with group elements much more.

I think earlier on...perhaps I would have found the group to be more enticing to me. I have found my community because I am a little bit further in on my cancer journey, so I have my community, so I didn’t need to engage much there.

age 50-59 years; metastatic disease

#### Recommendations From Oncologists (Most Powerful)

Participants felt that if their oncologist had recommended the app to them, they would have been likely to try it, and they linked this to having this recommendation earlier in their care pathway.

I ballooned during chemo, so if a doc had been like, “hey, do what you’ve gotta do to survive, but if you’re interested, this is an app that’s trying to help women maintain their underlying health while they’re doing cancer treatment, and including your mental health. That would have been powerful.

age 40-49 years; localized disease

### Desire for Weight-Focused Content Tailored to the Breast Cancer Experience

#### Too Much Focus on Emotional Content, Not Enough on Weight (Loss)

All participants had background knowledge of Noom (some had tried the Noom Weight app) and saw it as a weight loss program. They described that this had therefore been a key motivator for participation, and they expressed a strong desire to lose weight, which was often perceived as gained as a result of their cancer treatments. This led to an element of disappointment when the content of the HHC app was focused on psychological or emotional aspects of having breast cancer and weight maintenance.

I think of Noom as a weight loss tool, I was like oh great here’s this thing I’ve been meaning to try and hadn’t (be) cause of cost that I can try, tailored to me for weight loss.

age 40-49 years; metastatic disease

I feel like it spent a lot on emotional stuff -- maybe it should -- but I don’t know. I got a little grumpy about it.

age 40-49 years; localized disease

...my biggest thing going into it was I wanted to lose the weight, and at the very beginning it seemed to be more, more of that like self-acceptance, self-grace, that sort of thing, without ever getting into the – okay this is what we’re gonna do to make a change.

age 30-39 years; localized disease

This was true for participants with metastatic disease and those with localized disease. One participant described already having developed good coping skills after being diagnosed with an incurable disease, and what they really wanted from this app was weight loss support.

I live with this stage 4 diagnosis, my emotional, my coping skills are pretty strong– I didn’t get much out of them that helped me with my actual goal which was weight loss.

age 40-49 years; metastatic disease

However, participants did like the general idea of showing gratitude to and being kind to themselves (although most would like these aspects linked directly to weight loss). One participant with depression who had recently experienced a breakdown engaged more with the emotional support content (this participant did not engage with the behavior change elements and actually reported gaining weight during the study). However, the participant did perceive benefit from the emotional support.

Although most participants expressed that they were hoping for more focus on weight loss and that there was too much focus on the emotions associated with living with cancer, some did express that they found the psychological advice useful.

...feel like I’m able to more identify what the stressors are and kind of bring myself back a little bit.

age 30-39 years; localized disease

...there was one particular exercise where they talked about when these types of thoughts get in your head, to just use the terminology of, “stop it.” And that really hit home for me in a lot of different ways, because when I’m dieting, or really in any other aspect of my life, I tend to be an all-or-nothing-type person.

age 50-59 years; metastatic disease

#### Tailoring to the Breast Cancer Experience

While participants acknowledged a strong desire for an app to support weight loss, there was extensive discussion about the need to tailor the app content more to people with breast cancer. Many participants described how they felt the content was quite general, with very little mention of cancer in the content.

I know this was like for breast cancer participants, but I’m not sure if um there’s people who don’t have breast cancer that were doing it or using it as well but I thought that there would be more information on like, breast cancer and like medications, stuff like that.

age 30-39 years; localized disease

I guess because throughout the whole thing there was really not a lot of mention of cancer - it was more of everything else, so I really don’t feel like, I guess I feel like I was going into there thinking that this would be more a cancer-based thing, um the psychological effects of the cancer while losing weight, I don’t think it mentioned it hardly at all when I think about it.

age 50-59 years; localized disease

In particular, participants described how they would have liked more information on specific treatment side effects and guidance on appropriate health behaviors to deal with these (particularly, common active treatment side effects like nausea and diarrhea), in line with a desire for an app during active treatment.

If you could have talked about nutrition through chemo - like recipes, how to eat through nausea. For me, like I had some major surgeries, so like, “what would be good nutrition to focus on to rebuild after surgery” would have been helpful. I know that they talk a lot about how you have to focus on protein to help your body rebuild. And also -- maybe you say this somewhere in the app and I missed it.

age 50-59 years; localized disease

Some women also discussed the effect of hormone treatment on their weight and that they were seeking information on this, whether it is possible for them to lose weight while on this treatment, and how they can achieve this.

...the hormone therapy changes so many things, like your – how your metabolism runs, how you and your body will feel at different times of day and different things like that.

age 30-39 years; localized disease

I don’t actually know if its possible for women on hormone therapies to lose weight.

age 40-49 years; metastatic disease

### Tracking of Health Behaviors That are Generally Popular

#### Meal Logging and Color Coding of Diet Were Popular

Meal logging and color coding of diet were popular app features.

I really liked how like when you started typing things in it came up so I didn’t have to like put in the dietary information – like I literally just typed in like coffee or whatever and it came up, already had the calories and stuff in there. Um yeah they pretty much had everything I think there was just one thing that I had to like put in myself. Um that was, that was a good aspect of it I really liked that part.

age 30-39 years; localized disease

I did like having the breakdown of different colors of food because, you know, although I do eat healthy, I do eat -- I try to limit meat to one serving per day and then more plant-based, and it’s not like you can’t have plant-based proteins, but some things like nuts or seeds, you know, they’re red foods, so I think just being cognizant of, you know, if I have nuts, being like ‘am I eating one serving of nuts or am I taking a handful, which could be more than one?’ Like just those types of things; just trying to make sure the quality of my food remains high, despite if there’s foods in the red.”

age 40-40 years; localized disease

However, there was a perception that these could have been tailored more to the cancer community so that women could include information on the treatment they had or the side effects they were experiencing to understand how that was influencing their diet.

It might also be nice to be able to have like a note section, where you could be like -- for me, if I was having a certain digestive issue, I could put like, ‘this is what I ate, is my digestion good/bad, or a comment, or something’ in there.

age 40-40 years; localized disease

...you don’t have an option for, “did you have your infusion today? Are you nauseous today?” You don’t, like, talk about that treatment at all and how that’s impacting my ability to take in nutrition or what’s coming out of me.

age 50-59 years; localized disease

Participants engaged with the idea of having calorie targets, but one felt that they needed to be more individualized.

...even with this it was telling me I wasn’t eating enough calories when I was like stuffed full and gaining weight.

age 40-49 years; metastatic disease

#### Water or Fluid Tracking was Useful for the Cancer Community

A number of participants highlighted the tracking of water as useful and especially relevant to the breast cancer community as treatments can often lead to constipation or diarrhea, so sufficient fluid intake is particularly important.

I loved the water tracking and that’s also, again, for early-stage and metastatic, also by cancer trait, people tend to get a lot of diarrhea. They get heart disease, but everyone on hormone therapy, we all get constipated. I have to drink gallons of water a day to not have an impacted bowel, and so I loved the water reminders.

age 40-49 years; localized disease

#### Physical Activity Tracking was Very Useful, but the Step Target was Too Low

Tracking of activity was popular and consistently perceived as useful. This was seen as especially useful when participants experienced a change in their daily routines that resulted in a reduction in steps taken in their daily lives, either due to their initial diagnosis and treatment or due to the COVID-19 pandemic.

I just think tracking is really useful. You know, I used to be more active than I am now. I used to walk my kids to school and it was a half mile from my house and bike to the grocery store, and I sometimes forget that I'm not getting all of this activity just from being alive anymore like I used to, so tracking it has me out walking my neighborhood while we’re on the phone. I mean I've gotten like 2,000 steps since we’ve been on the phone, and tracking it has me doing this.

age 40-49 years; localized disease

However, there was a general perception that the step count was pitched too low. A number of participants talked about being surprised by their “2000 steps” target, feeling that this was not challenging enough, which was then not motivating, and they disengaged from this aspect of the app. Some discussed how this might have been more reasonable during active treatment but that the targets set in the app should be based on the individual’s initial step count. Some also referenced information that they were aware of in the public domain, like step count targets, which influenced their perceptions.

...it started I believe at 2000 steps a day which is like super low so I wasn’t sure, um, you know why that starts so low um but I think it raised to like 4000 or whatever but I think like the standard like what how many steps you’re supposed to take in a day is 10,000 which is like the universal.

age 30-39 years; localized disease

#### Increased Prominence of Physical Activity Preferred

Participants in this sample had a high awareness of the importance of physical activity after a cancer diagnosis and felt that this should have had more prominence, with a view that evidence-based information about physical activity after cancer should be provided.

...since I had a double mastectomy...now I can barely do a push up so maybe something information on, what exercises I could be doing to like help get my muscle back.

age 30-39 years; localized disease

Some participants discussed highlighting cancer-specific guidelines.

I don’t know how cancer-specific you want to get in this app, but it would have been interesting: “the latest research shows:...” you know? And, actually, for people with metastatic disease, people who exercise can have better overall survival, you know.

age 40-49 years; localized disease

#### Medication Adherence Feature Not Used

No participant used the medication adherence or logging feature. They described how they came to the app with strategies already in place (like pill boxes) and did not perceive a need or desire for this feature.

I never like notified it but it was always there, but “did you take your medicine today”, I think when I saw it I hid it, but I’ve got a pretty good routine set up, cause I take my medicine a couple times a day, so that – that I didn’t feel like I really needed.

age 30-39 years; localized disease

### High Value of In-App Social Support

#### Coaches Provided Accountability and Increased Engagement

Participants were very positive about their one-to-one coach. They felt supported without being judged, and making plans with their coach gave them a sense of accountability and kept them engaged with the app.

I liked the amount of checking in she did with me, it wasn’t overbearing, it wasn't too much, and she just encouraged me to keep up with the articles and things like that.

age 50-59 years; metastatic disease

She just kept me focused and digging deeper.

age 50-59 years; localized disease

I would say ok my goal this week is like, I’m gonna walk every day and um she would be like ok well ’I’ll check in with you on Friday, so like the fact that like there was some kind of accountability mechanism, um I really like that.

age 40-49 years; metastatic disease

However, 2 participants chose not to engage with their coach as they felt they did not need this form of support.

#### Groups Were Popular

In general, groups were viewed as a positive feature, and the ability to engage or not as desired was appreciated. Some participants acknowledged that they just were not “group” people but still felt it was a useful feature to have available.

...basically people telling stories about their lives and what works and what doesn’t work for them, so that’s like my favorite model of gaining wisdom about the human condition. I think it’s because I’ve been doing it for so long, and people have interesting ways that they try to get more steps in and connecting and stuff like that.

age 40-49 years; localized disease

One participant commented on group size, suggesting that they had really enjoyed an initial small group that was then merged with a larger one, including people who were much more established, and the participant felt less comfortable in this larger group.

I really like the idea of the group and I felt like the group was very weight loss focused which I liked, I had a small group then got combined with another group …and it was also like fifty people which made me feel pretty exposed about being vulnerable, like I would feel more comfortable in a group of 10 to 15 but that may be hard when people aren’t actually posting.

age 40-49 years; metastatic disease

There were some interesting discussions around the presentation of only positive outcomes (especially around weight loss), and how this could be disheartening if someone was not losing weight. For example, 1 participant mentioned that they had gained a little weight, and after announcing this in the group, the participant received a private message from another participant saying they had too but felt uncomfortable sharing. The participant suggested that this could be mitigated by encouraging people to share when things had not gone so well and for others to empathize and share solutions.

I had a couple people contacted me, one on app and two off app, (be)cause the breast cancer community is small so folks knew who I was to say that they had – they didn’t feel comfortable posting publicly that they had also gained weight and were pretty upset about it.

age 40-49 years; metastatic disease

#### Mixed Views on Including Early Stage and Metastatic Disease Together

There was an interesting juxtaposition between those with early and metastatic disease on the benefits of mixing people with different cancer stages in groups. Participants with earlier-stage disease felt anxious and self-conscious about discussing certain things like “getting back to normal” as they were concerned about upsetting those with metastatic disease.

I got a little anxious in the group. I think most of us had early stage disease, but there was a woman with metastatic disease, and I feel, I become very anxious about being insensitive with someone who has metastatic.

age 40-49 years; localized disease

In contrast, 1 woman with metastatic disease really appreciated being included in groups with those with earlier-stage cancers and felt this was a much needed opportunity to break down barriers.

I really really appreciated that this app put people together that were both metastatic and early disease, but I’m involved in a cancer advocate program, and I chose the young advocate program because I really believe we need to break down barriers between early stage and late-stage people.

age 40-49 years; metastatic disease

This participant expressed that she was really pleased that women with metastatic cancer were being included in this research and that the app was designed for them as well as those with early stage disease.

I know there are women who have better response to their therapy who will probably – there are more and more women who are living 10-15 years with metastatic breast cancer and yet there’s a very little for us because the discourse is still meant assuming we’re all gonna be dead in two years, which of course many many women still are, but not all of us.

age 40-49 years; metastatic disease

## Discussion

### Summary of the Findings

This early phase user experience study found that an app-based intervention with peer and coach support was an acceptable and appealing method of self-management for women after breast cancer diagnosis. However, participants expressed that they would have found this most helpful early in their cancer journey because they had already instilled lifestyle habits after active cancer treatment, and their use of the app declined over the 4 weeks, implying that it became less useful to them over time. The areas in which they particularly wanted support were managing the side effects of their treatment and behaviors that would lead to weight loss. They mostly valued the “emotional” content when this was linked to their weight management behaviors and highlighted that they were looking for content that was more tailored to women with breast cancer and dealt with challenges specific to this group.

### Comparison With Other Studies

Participants expressed a strong desire for weight management support. This may have been particularly prominent in this study because of their prior knowledge of Noom as a weight loss program. However, it is in line with previous work that has shown that weight gain after diagnosis is a source of concern in many women with breast cancer and that those living with and beyond cancer want to focus on their behaviors in order to play an active role in their health management [6,7,17,32]. In addition, weight gain, overweight, and obesity are associated with worse treatment side-effects and higher mortality after breast cancer diagnosis [33], so it is encouraging that the women we interviewed viewed an app-based intervention as an appealing method of accessing support with weight management. While the previous literature focuses mainly on early stage breast cancer, in our study, patients with metastatic disease also felt strongly that they desired weight or self-management programs and behavioral support. This reflects the changing landscape of treatment since patients with metastatic disease can live for many years; thus, future research should consider how to support those with metastatic disease to live as well as possible [34,35].

In this study, participants reported that they would have preferred the app content to be more tailored to the specific needs of those living with and beyond breast cancer, and the desire for tailoring in apps is consistent with other qualitative studies exploring app preferences [13,22,23]. This presents a challenge in terms of design and cost, and highlights the need to involve relevant cancer specialists in the design of apps intended for this population [16]. The strength of an app-based intervention is that it can successfully tailor a program to the end user, and further development of the program will consider how this could be incorporated. It is exceptionally challenging to develop dietary recommendations that not only encompass general healthy eating guidelines but also account for treatment side effects. However, app content could be continually updated to incorporate the newest evidence, and eventually, back-end data of dietary tracking from the cancer app could help inform tailoring algorithms.

In line with previous research, a recommendation to use the app from oncologists was considered to be most valued, and although health care professionals have reported a lack of confidence and time to provide health behavior support [13], they may feel able to recommend an app to participants that can provide what their patients are looking for. This would fit with reported views that the delivery of behavioral advice should be framed as part of treatment and must be cost-effective [9].

The overall style of the app was popular with participants. They particularly liked the tracking features and the one-to-one coaches. Self-monitoring is known to be an important tool in weight management [36], and there are many apps that can be used for this, but they vary in their usability [37]. Interacting with the one-to-one coaches gave participants a sense of accountability as they felt someone was monitoring whether they acted on their plans and goals. Previous work has also highlighted the importance of this component of interventions in this group [38].

Our previous work suggested that social support was a desired or important feature [13,23], and this was supported by most participants in this study. Discussion groups with other women living with and beyond breast cancer also provide accountability as well as social support, encouragement, and ideas for ways to manage thoughts and behaviors [36]. These were used more by some participants than others, but it was generally agreed to be a useful app feature. Some challenges of these groups were raised. These included a feeling of not being able to discuss perceived failures in weight management. This could be a feature of the particular group that these participants were in, but it does highlight that apps that use a group coach could include coaching that encourages the sharing of setbacks. Some of the participants with localized disease were concerned about sharing their feelings with women with metastatic disease because they felt it might be insensitive, but the women with metastatic disease really valued being included and felt that this was important. As some women with localized disease will go on to have metastatic disease, building relationships in this group would potentially be helpful; however, more work is needed to find ways to meet the needs of both groups of women.

Participants in this study felt that physical activity needed to be more prominent in the app as they were aware of the benefits that this can have. Increasing physical activity is something that women can do to improve their mental and physical health and is something that they can control at a time when things can feel out of control [39]. Participants seemed to be using the automatic adjustable step targets rather than setting their own targets and found these to be too low. This suggests that the option to overwrite this with your own targets needs to be highlighted in the app and that the starting point for automatic targets needs to be better individualized. Participants were happy to track their calories, and only one participant expressed concerns about the calorie target (the participant felt it was too high). It could be further explored whether app users should be able to adjust the calorie target or if coaches could do this for participants. The medication adherence feature was not used, but this might have been because these women already had systems in place to take their medications. As medication adherence can be suboptimal [40], more work is needed to see if this would be a helpful feature for those closer to their diagnosis when they were first taking their medications.

This study adds to previous work suggesting that there is interest in apps that support women with breast cancer [15-17] and highlights that what these women wanted most was help with weight management that is specifically tailored to the breast cancer experience. The themes identified could be used to develop this intervention to better fit these needs and to inform other interventions for this population, as user feedback is an essential component in intervention development [16].

There are however some limitations of this study. In particular, this was a small sample of predominantly white, married, and employed women who were interested in trying out this app, and therefore, the findings may not be transferrable to women from other sociodemographic groups. Findings related to the views of patients with metastatic cancer are potentially important. However, only 2 participants in our sample had metastatic disease; thus, more work investigating the views of this group is crucial. The interviews were conducted by a member of Noom, which could have introduced some bias. However, participants were made aware that this was a very early prototype and that we wanted to understand positive and negative views in order to make changes before further development (and many suggestions for changes were indeed made in interviews). This work was conducted as an industry-academic partnership. This benefitted both sides of the partnership. The industry team benefitted from the expertise of the academics in conducting rigorous high-quality research. The academics benefitted from the opportunity to understand user experiences of a high-quality app, with a higher likelihood of this research being taken forward than when working independently of the industry.

### Conclusion

This early user experience work showed that women with breast cancer found an app with integrated social and psychological support appealing to receive support for behavior change and weight management or self-management. However, many features were recommended for further development. This work is the first step in an academic-industry collaboration that would ultimately aim to develop and empirically test a supportive app that could be integrated into the cancer care pathway. Although further work is required, the results suggest that this approach has the potential to make an important contribution to cancer care for this group.

## Appendix

Multimedia Appendix 1 Interview schedule.

## Abbreviations

HHC: Healthy Habits after Cancer
