# Supplementary material for: Prototype of an App Designed to Support Self-Management for Health Behaviors and Weight in Women Living With Breast Cancer: Qualitative User Experience Study
Source: JMIR Cancer. 2024 Dec 20;10:e48170. doi: 10.2196/48170 (PMC11699501; doi:10.2196/48170)
Supplement: Multimedia Appendix 1 [file cancer_v10i1e48170_app1.pdf]

Supplementary Data: Interview schedule

1. Firstly, can you tell me a bit about yourself?
    a. Where are you in your cancer journey?
    b. What made you want to volunteer for this study?

2. Now can you talk me through your experience of using the app?
[Prompts for each part of the app if they don't cover naturally]

- Meal logging
- 1:1 coach/goal specialist
- Group & group coach
- Exercise/physical activity logging
- Medication reminders
- Reading articles

3. Check that they used all of the features (prompt the interviewee with the list) and if there were bits they didn't use ask them about why?

4. What articles or topics did you particularly like? Find the most helpful?
    a. What articles or topics did you dislike? Find the least helpful?
    b. Specific features (listed above in italics) or BCTs?
    c. Were there topics that were not addressed (in the articles) that you would have liked to have addressed or learned more about?

5. Was there anything that made you hesitate toward using the app? (For example, the time commitment, unsure about how helpful it would be, not feeling like you had the need for it at the moment, etc.)

    If so, how did you overcome this?

6. How appropriate and/or relevant was the app for your personal circumstances?

7. What changes (for example, behaviors, ways of coping) have you made in your life due to using the app? What do you think was the most important or helpful change?

8. Have you noticed and changes in your physical health from using the app? . What do you think was the most important or helpful change?

9.Have you noticed changes in your mental or emotional health since have you noticed from using the app?

    What do you think was the most important or helpful change?

10. Looking back, do you think there is anything you could have done differently to get more out of the app?
    a. How do you think where you are in your cancer journey affected how much you used the app?
    b. How much have you benefited from the app?
    c. How do you imagine your use of the app might change/have changed over the course of your cancer journey? [basically asking whether if they had used it closer to their diagnosis, during treatment, later on in survivorship, etc. if that would have made any difference in how helpful they found it to be.]

        If so, what changes would you make?

11. Do you think the app could be improved for people with breast cancer who are on hormone therapy?

Was there anything that was missing that you would have liked to see in the app?

12. Who should direct people with breast cancer to a breast cancer-specific app? (e.g., oncologist, primary care physician, etc).

a. Who do you think should refer women to use the app?
b. Who could have recommended the app to you that you would have listened to the most /would you be most likely to listen to and to ultimately try the app? [see examples below]
c. How likely would you use this app if it were referred or suggested to you by someone vs. you deciding to try it on your own? .For example,

- Your cancer healthcare provider (oncologist, radiologist, surgeon, advanced practice clinician)?
- Your general healthcare provider, like a primary care physician?
- Your / a mental health professional (psychologist, counselor, social worker)
- A friend or peer?
- A family member?

13.When in the cancer journey should the use of this kind of app be discussed and promoted to patients? (e.g., at time of diagnosis, conclusion of chemo, etc)

14. Any other comments or feedback regarding the app?
